# A pH-induced conformational switch in a tyrosine kinase inhibitor identified by electronic spectroscopy and quantum chemical calculations

**DOI:** 10.1038/s41598-017-16583-z

**Published:** 2017-11-24

**Authors:** Muhammad Khattab, Feng Wang, Andrew H. A. Clayton

**Affiliations:** 1 Centre for Micro-Photonics, Faculty of Science, Engineering and Technology, Swinburne University of Technology, Melbourne, Victoria, 3122 Australia; 2 Molecular Model Discovery Laboratory, Department of Chemistry and Biotechnology, Faculty of Science, Engineering and Technology, Swinburne University of Technology, Melbourne, Victoria, 3122 Australia; 3 School of Chemistry (Bio21 Institute), University of Melbourne, Parkville, Victoria, 3052 Australia; 4 School of Physics, University of Melbourne, Parkville, Victoria, 3052 Australia

## Abstract

Tyrosine kinase inhibitors (TKIs) are a major class of drug utilised in the clinic. During transit to their cognate kinases, TKIs will encounter different pH environments that could have a major influence on TKI structure. To address this, we report UV-Vis spectroscopic and computational studies of the TKI, AG1478, as a function of pH. The electronic absorption spectrum of AG1478 shifted by 10 nm (from 342 nm to 332 nm) from acid to neutral pH and split into two peaks (at 334 nm and 345 nm) in highly alkaline conditions. From these transitions, the pKa value was calculated as 5.58 ± 0.01. To compute structures and spectra, time-dependent density functional theory (TD-DFT) calculations were performed along with conductor-like polarizable continuum model (CPCM) to account for implicit solvent effect. On the basis of the theoretical spectra, we could assign the AG1478 experimental spectrum at acidic pH to a mixture of two twisted conformers (71% AG1478 protonated at quinazolyl nitrogen N(1) and 29% AG1478 protonated at quinazolyl nitrogen N(3)) and at neutral pH to the neutral planar conformer. The AG1478 absorption spectrum (pH 13.3) was fitted to a mixture of neutral (70%) and NH-deprotonated species (30%). These studies reveal a pH-induced conformational transition in a TKI.

## Introduction

Determination of acid dissociation constant (pKa) of drugs gains paramount significance from the perspective of dosage form formulation, pharmaceutical analysis, and studying drug pharmacokinetics^1,2^. Drug solubility, lipophilicity, protein binding and membrane permeability are also influenced by its pKa value^3^. For instance, basic drugs with pKa > 7.4 (blood pH) are ionized displaying slower diffusion rates across cellular membranes^2^. Hence the drug ionization constant is one of its very important physicochemical properties.

Several techniques^4^ have been used for pKa determinations such as potentiometric titration^5,6^, UV-Vis spectroscopy^7,8^, reverse-phase high performance liquid chromatography^9^, and capillary electrophoresis^10^. UV-Vis spectroscopy takes advantage over other techniques since it is accurate, precise, reproducible and cost-effective using only micromolar concentrations of samples. It has been used for exploring electronic properties of the ground and excited states of fluorophores^11,12^. It has helped in studying physicochemical phenomena like FRET^13^, proton transfer^14,15^ and solvatochromism^16,17^. The two prerequisites for successful determination of pKa by UV-Vis spectrophotometry are a) presence of chromophore near to ionization centre and b) change in absorbance spectrum as a function of compound ionization^2^. For this reason, optical pH probes have gained a wide range of applications in analytical and biomedicinal chemistry^18^. These probes have been used for measuring intracellular pH^19,20^ and monitoring blood pH^21^. Such probes are cornerstone for the development of chemical sensors used in cell biology, biomedical diagnostics and environmental monitoring^22,23^.

Numerous studies reported that the protonation pattern of a chromophore can affect its UV-Vis absorption and fluorescence spectrum^7,8,24^. Protonation in some cases causes a bathochromic shift of absorption maxima with varying optical densities of absorbance bands^25–27^. Accordingly, the acid-base properties of a chromophore can be evaluated by means of absorption/fluorescence spectroscopy^24^. A number of theoretical studies have also been performed to investigate the protonation processes^28,29^, the electronic and geometric structures of the excited prototropic states^30–32^ and protonation microequilibria^33,34^.

Tyrosine Kinase Inhibitors (TKIs) are organic compounds showing anti-proliferative activity against cancer cells^35^. In the last two decades, extensive research has been conducted to develop new generations of selective TKIs with higher potency and resistance to tyrosine kinase mutations^36–38^. 4-anilinoquinazoline-based TKIs have been intensively studied, leading to a number of FDA-approved drugs such as Afatinib^39^, Erlotinib^40^, Gefitinib^41^, Lapatinib^42^, and Vandetanib^43^.

AG1478 is one of the tyrosine kinase inhibitors^44,45^ besides being a potential DNA intercalating agent^46^. It inhibits cell growth through binding to epidermal growth factor receptor. Preclinical and clinical studies showed its selectivity and efficacy to inhibit hepatocellular carcinoma^45^, autocrine growth in human lung and prostate cancer cell lines^47^, cisplatin-resistant human lung adenocarcinoma^48^, and proliferation of nasopharyngeal carcinoma CNE2 cells^49^. Studies on active pharmaceuticals which bind to cell DNA emphasised that the cationic form of a drug intercalates with DNA bases more strongly than neutral species, while the anionic form of a chromophore is a poor intercalating agent due to the columbic repulsion between negatively charged DNA backbone and drug^50–52^.

**Figure 1:**
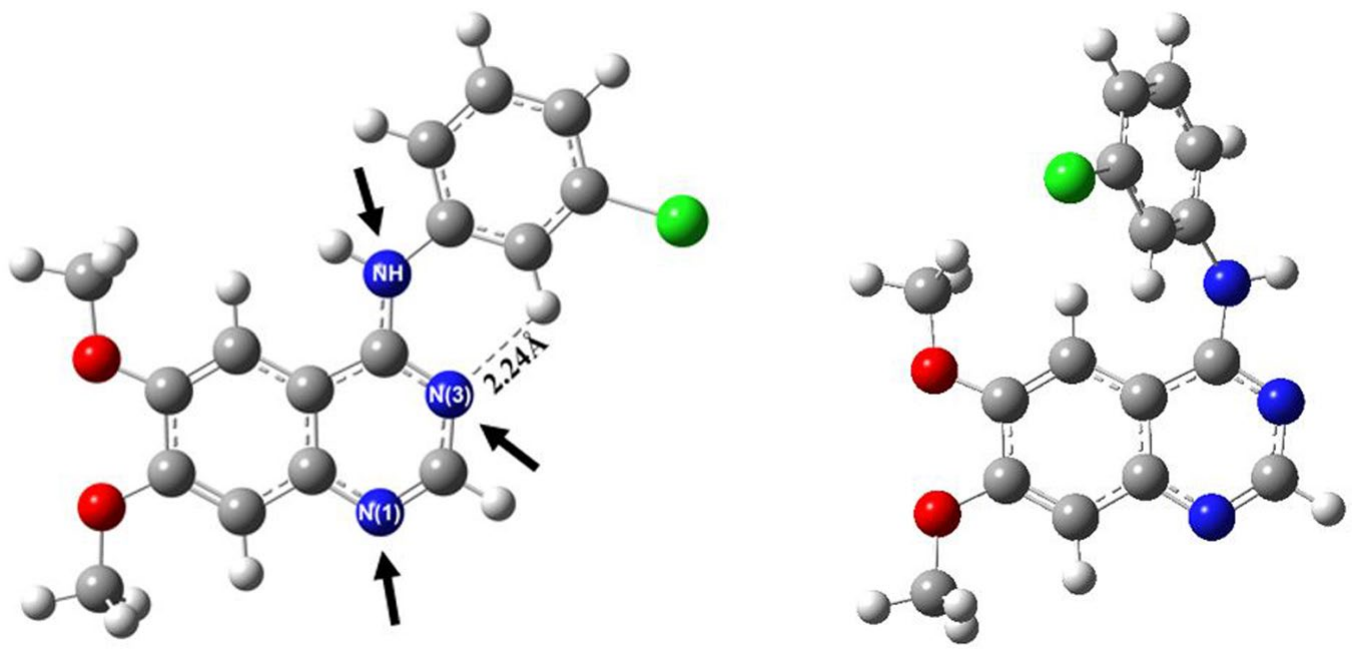
Molecular structure of AG1478 in its planar and twisted conformations^55^. White, grey, blue, red and green colors of atoms denotes to hydrogen, carbon, nitrogen, oxygen and chlorine atoms respectively. Dashed line and label refers to the intramolecular hydrogen bond and its length.

Our own studies on AG1478 have revealed that the spectroscopic properties are sensitive to both environment^53,54^ and AG1478 conformational state^55^. For example, two conformers- one planar and the other twisted- were identified based on quantum chemical calculations and experimental absorption spectra^55^. Structures of AG1478 in its planar and twisted conformations are depicted in Fig. 1. Two nitrogens on the quinazoline ring are denoted N1 and N3, respectively, while the aniline ring amino moiety is denoted by NH linker.

However, the detailed optical properties and electronic structure of prototropic forms of AG1478 have not been reported so far. In this article, we probe the UV-Vis spectral properties of AG1478 as a function of solution pH. A theoretical investigation of protonated, neutral and deprotonated forms of AG1478 is also performed. Based on our theoretical calculations, we assign the observed AG1478 spectra gaining insights into the geometry and electronic excitations of the prototropic forms of AG1478. We discuss the implications of our findings to drug pharmacodynamics.

## Results

### Absorption spectroscopy study

**Figure 2:**
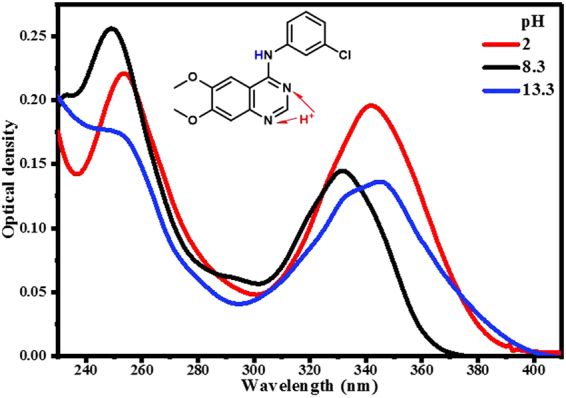
Representative absorption spectra of AG1478 in aqueous buffered solutions at pH 2, 8.3 and 13.3.

Absorption spectra of AG1478 in different pH-buffered solutions (pH 0.3 to 13.3) were measured. Figure 2 depicts selected absorption spectra for AG1478 at pH 2, 8.3 and 13.3. The recorded absorption spectra in strongly acidic conditions at pH 0.3–2.3 (0.3, 0.6, 0.9, 1.2, 1.6 and 2) exhibited a prominent peak in the 300–400 nm region with λ_max_ at 342 nm. Spectra of AG1478 in alkaline solutions at pH 7.3–12 had relatively reduced optical densities in the 300–400 nm region compared to acidic conditions and were shifted to the blue with a λ_max_ at 332 nm. The highly alkaline solutions of AG1478 (pH 13.3) displayed two overlapping peaks at 334 nm and 345 nm with enhanced absorbance in 380–400 nm region with respect to AG1478 spectrum at pH 8.3, as indicated in Fig. 2.

**Figure 3:**
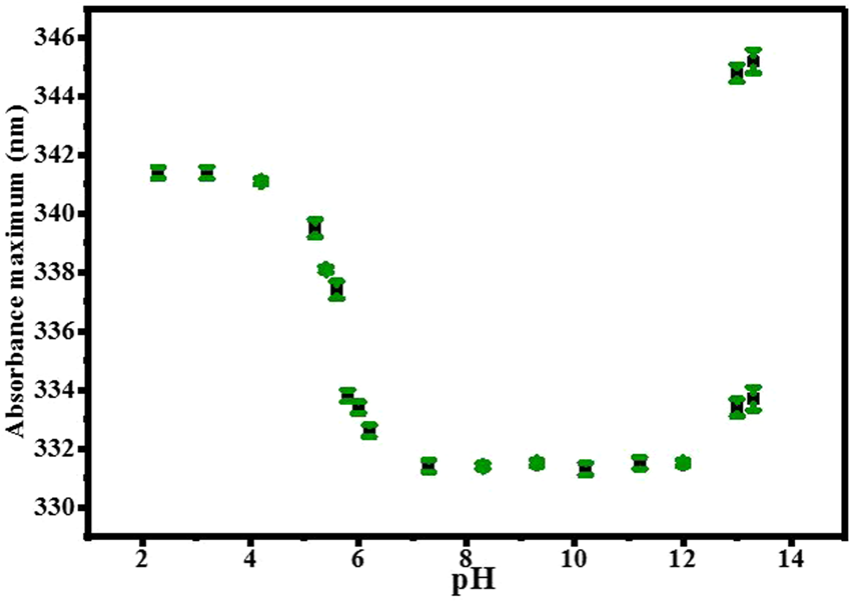
Wavelength absorption maxima in 300–400 nm region of AG1478 in aqueous buffered solutions at pH ranging from 2.3 to 13.3. Values were determined from the average of five scans. Error bars indicate 95% confidence interval widths.

A plot of the longest wavelength absorption peak of AG1478 in all studied buffer solutions is depicted in Fig. 3. The absorbance maximum of AG1478 was *ca*. 342 nm in pH 0.3–2.3 buffered solutions. A hypsochromic shift of 0.0 ± 0.4 nm, 0.3 ± 0.3 nm and 1.6 ± 0.4 nm was observed by changing pH 2.3 to 3.2, 3.2 to 4.2 and 4.2 to 5.2, respectively. This systematic blue shift continued reaching its maximum at pH 7.3 (10.0 ± 0.4 nm, relative to pH 2.3), refer to Fig. 3. The absorption maximum was then constant at *ca*. 332 nm over the pH range of 7.3–12. The broad absorption band of AG1478 at pH 13 and 13.3 showed two absorption maxima at *ca*. 334 nm and 345 nm.

**Figure 4:**
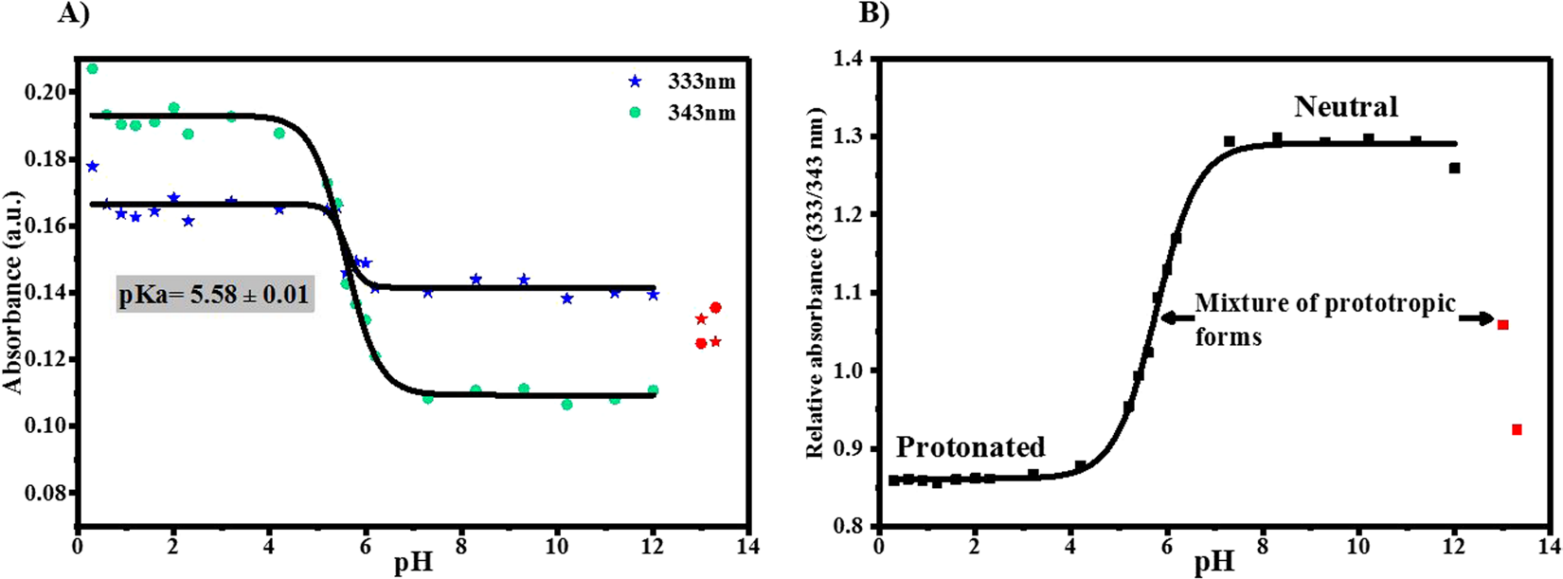
(**A**) Plot of influence of solution pH on the optical density of AG1478 at 333 nm and 343 nm and (**b**) Plot of 333/343 nm absorbance ratio as a function of solution pH. Data points (marked red) at pH 13 and 13.3 were excluded from Boltzmann function fit.

A plot of the optical density of AG1478 at 333 nm and at 343 nm as a function of solution pH is illustrated in Fig. 4a. The 333 nm and 343 nm wavelengths were selected as a measure for the absorbance due to the planar and twisted conformers, respectively. At both wavelengths, the optical densities remained relatively constant in the pH range 1–4, decreased in the pH range 4–7 and then plateaued from pH 7–12. These features are consistent with an equilibrium transition from one protonation form to another. Using a Boltzmann function to fit the data in the pH range 1–12, we extracted a pKa of 5.58 ± 0.01 for the AG1478 molecule. Further changes to AG1478 optical density at the very end and beyond the practical pH scale *viz*. pH ≥ 12 and pH ≤ 0.6 can be seen in Fig. 4a, but these values were not analysed further. We also plotted the change in ratio of AG1478 absorbance at 333 nm to 343 nm as a function of pH, Fig. 4b. The transition at acid pH is very clearly visible from this plot as well as a second transition at pH > 12. The pKa values extracted from Fig. 4b (pKa AG1478 = 5.7) agreed well with the values obtained from Fig. 4a (pKa AG1478 = 5.6).

To summarize, it is clear that the protonation state of AG1478 influences significantly its spectroscopic properties. However based on the experiments alone it is not possible to assign the spectra to specific species. The changes in AG1478 spectral properties upon changing solution pH could be attributed to a proton-induced change in AG1478 conformation and/or a change in AG1478 electronic configuration. Therefore, we aim to computationally explore the geometrical structures and vertical excitation energies of neutral and various ionic species of AG1478 in the next section.

### Computational study

**Figure 5:**
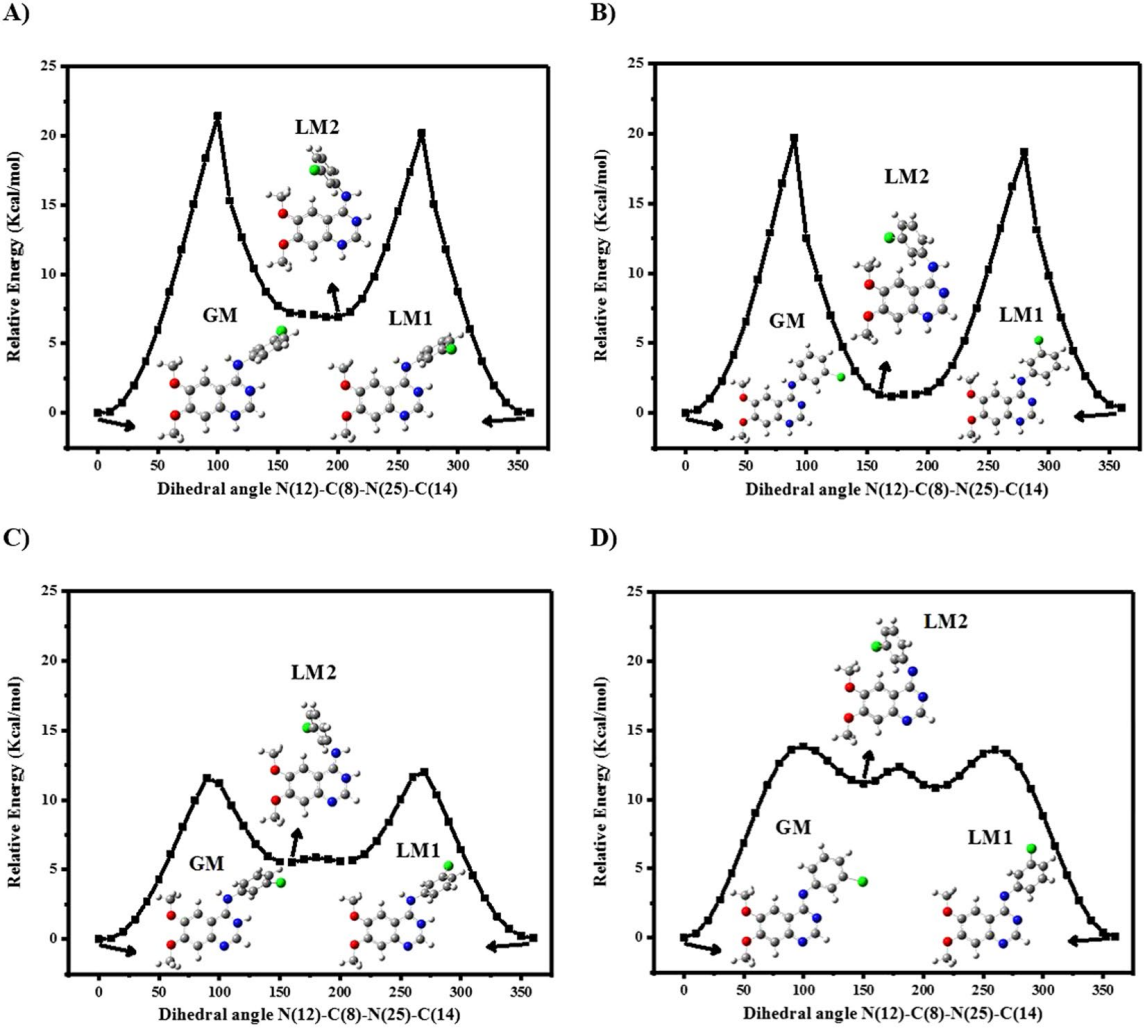
Potential energy surface scan of (**a**) ^+^N(1)H^+^N(3)H, (**b**) ^+^N(1)H, (**c**) ^+^N(3)H and (**d**) deprotonated structures of AG1478 at B3LYP/6-31 G in vacuum. Arrows refers to the 3D structure of AG1478 at a corresponding point on PES surface. White, grey, blue, red and green colors of atoms denotes to hydrogen, carbon, nitrogen, oxygen and chlorine atoms respectively.

In our theoretical study, we considered five prototropic structures of AG1478. We performed potential energy surface (PES) scan for four AG1478 structures in gas phase, diprotonated at N(1)N(3), monoprotonated at each N(1) and N(3) and deprotonated at NH linker as shown in Fig. 5a,b,c and d. PES scan of neutral form of AG1478 was published earlier by Khattab *et al*.^55^ All PES scan plots are depicted in Fig. 5 together with 3D structures of the global minimum and local minima.

PES of diprotonated AG1478 (Fig. 5a **)** showed one global minimum with calculated energy difference of ≈7 kcal/mole with the second local minimum. All diprotonated structures had anilino group twisted relative to quinazolyl moiety.

The PES of the protonated AG1478 at N(1) exhibited a global minimum and two local minima with a very small energy difference (≈2 kcal/mole), as shown in Fig. 5b. Unlike the diprotonated species, the global minimum structure of protonated AG1478 at N(1) had the quinazoline and aniline rings in a planar conformation. Similar to the diprotonated form, the aniline group was twisted relative to quinazoline ring in the highest energy local minimum.

The PES of AG1478 monoprotonated at N(3) had a global minimum and two local minima with a nearly similar energy difference to the PES di-protonated at N(1)N(3) (≈6 kcal/mole). The two local minima at step 17 and 21 were almost identical and therefore we attributed these to one structural form of AG1478. All protonated structures at N(3) were identified as twisted conformers similar to N(1)N(3) protonated structures as indicated in Fig. 5c.

In case of deprotonated structure of AG1478, PES scan showed two higher energy local minima than the global minimum (>10 kcal/mole) as can be seen in Fig. 5d. Minima at step 16 and 22 are almost structurally similar, therefore one of them was considered. Deprotonated AG1478 were identified as planar, pseudo-planar and twisted structures as calculated for monoprotonated N(1) structures.

The global minimum and the first local minimum structures in Fig. 5b and d were planar and pseudo-planar conformers respectively. Generally, planar conformations of AG1478 is energetically favoured when a proton at N(1) site is added or abstracted. However, addition of proton to N(3) site results in breakage of intramolecular H-bond responsible for maintaining structural coplanarity and extra stability of planar conformation over the twisted one. Therefore, N(3) protonated structures adopted only twisted configurations.

Global minima (GM) and local minima (LM) geometries were re-optimised using B3LYP/6-311+G* model and dielectric constant of water. A list of molecular coordinates of all studied structures is in Supplementary Materials Table S1. We calculated the binding energy for formation of prototropic states of AG1478 which are listed in Table 1. The proton binding energy was calculated by subtracting summation of total molecular energy (incl. zero point energy correction) of global minimum structure of neutral AG1478 and no/one/two proton(s) from total molecular energy of deprotonated/monoprotonated/diprotonated structures respectively.

**Table 1:** The proton binding energy values (kcal/mole) of ionic forms of AG1478 in water at B3LYP/6-311+G*.

Structure	GM	LM1	LM2
^+^N(1)H^+^N(3)H	−315.99	−315.77	−311.54
^+^N(1)H	−168.67	−168.47	−165.79
^+^N(3)H	−164.99	−164.99	−161.07
Deprotonated	292.20	292.22	297.26

*GM, global minimum; LM, local minimum.

Formation of diprotonated structure was theoretically calculated as the most favourable prototropic structure of AG1478. The maximum energy difference between minima structures was estimated <5 kcal/mole. Binding energy values exhibited small energy difference between monoprotonated AG1478 structures with energetic preference to N(1) protonation. A small energy gap (<4 kcal/mole) between different minima was revealed suggesting easy interconversion between conformers of same protonated structures. Also, the energy difference between different minima of ^+^N(1)H and ^+^N(3)H structures was less than 8 kcal/mole. In contrary, deprotonation of NH linker required spending energy (endothermic process). The order of structural stability based on binding energy calculations is as follows ^+^N(1)H^+^N(3)H > ^+^N(1)H > ^+^N(3)H > deprotonated.

An earlier study on quinazoline moiety is in line with our results. Sawunyama and coworkers calculated the proton binding affinity to each nitrogen of quinazoline^56^. Results revealed that protonation proceeds at any of quinazolyl nitrogens with binding affinity difference by 0.1–1.3 kcal/mole depending on applied model. Diprotonation at two nitrogens had a greater binding energy than one bound proton. It was concluded that quinazoline protonation proceeds as follows dication ≫ N3 monocation > N1 monocation. In contrary to our results, N3 monocation is slightly energetically favoured than N1 counterpart^56^. It might be due to the lack of 4-substituent group which would alter electron density of quinazoline ring.

Absorption spectra of protonated AG1478 were calculated deploying TD-DFT and the dielectric constant of water (*ε* = 78.35) in CPCM model. Complete spectra of various prototropic forms of AG1478 are depicted in Supplementary Materials Fig. S2. The lowest excitation energy transitions along with the corresponding oscillator strength and molecular orbital (MO) transitions are listed in Table 2. The maximum wavelength of the lowest lying electronic transitions of AG1478 ranged from 322 to 386 nm, depending on protonation state and conformation, however the number of transitions and MO contribution also varied to some extent. In contrary to all other studied structures, the diprotonated form exhibited two transitions in 300–400 nm region where HOMO → LUMO contributed to the lowest energy transition (S_0_ → S_1_) at 355–364 nm and HOMO → LUMO+1 and HOMO-2 → LUMO contributed to the higher energy transition (S_0_ → S_2_) at 321–327 nm. For the monoprotonated, neutral and deprotonated structures, HOMO → LUMO transition was the main contributing transition for only one electronic transition as indicated in Table 2.

**Table 2:** Wavelength, oscillator strength and molecular orbital transition contribution of the longest wavelength excitation bands in the 300–400 nm region for AG1478 in water using B3LYP/6-311+G* model. Only transition contribution > 10% is considered significant and listed in the table.

Structure	Exc. E (nm)	Osc. str.	No. of transitions	Transition contribution
N1N3-GM	355 321	0.3279	2	H→L (91%)
		0.1346		H→L+1 (79%), H-2→L (16%)
N1N3-LM1	356 322	0.3335	2	H→L (92%)
		0.1373		H→L+1 (81%), H-2→L (15%)
N1N3-LM2	364 327	0.2010	2	H→L (89%)
		0.1435		H→L+1 (76%), H-2→L (15%)
N1-GM	363	0.7613	1	H→L (98%)
N1-LM1	356	0.6740	1	H→L (98%)
N1-LM2	347	0.3198	1	H→L (96%)
N3-GM	322	0.2761	1	H→L (91%)
N3-LM1	326	0.3103	1	H→L (92%)
N3-LM2	347	0.2828	1	H→L (95%)
Neut-GM	331	0.6556	1	H→L (97%)
Neut-LM	337	0.3158	1	H→L (96%)
De-GM	371	0.7830	1	H→L (98%)
De-LM1	368	0.6127	1	H→L (98%)
De-LM2	386	0.2769	1	H→L (97%)

*GM, global minimum; LM, local minimum; H, HOMO; L, LUMO; Neut, Neutral.

**Figure 6:**
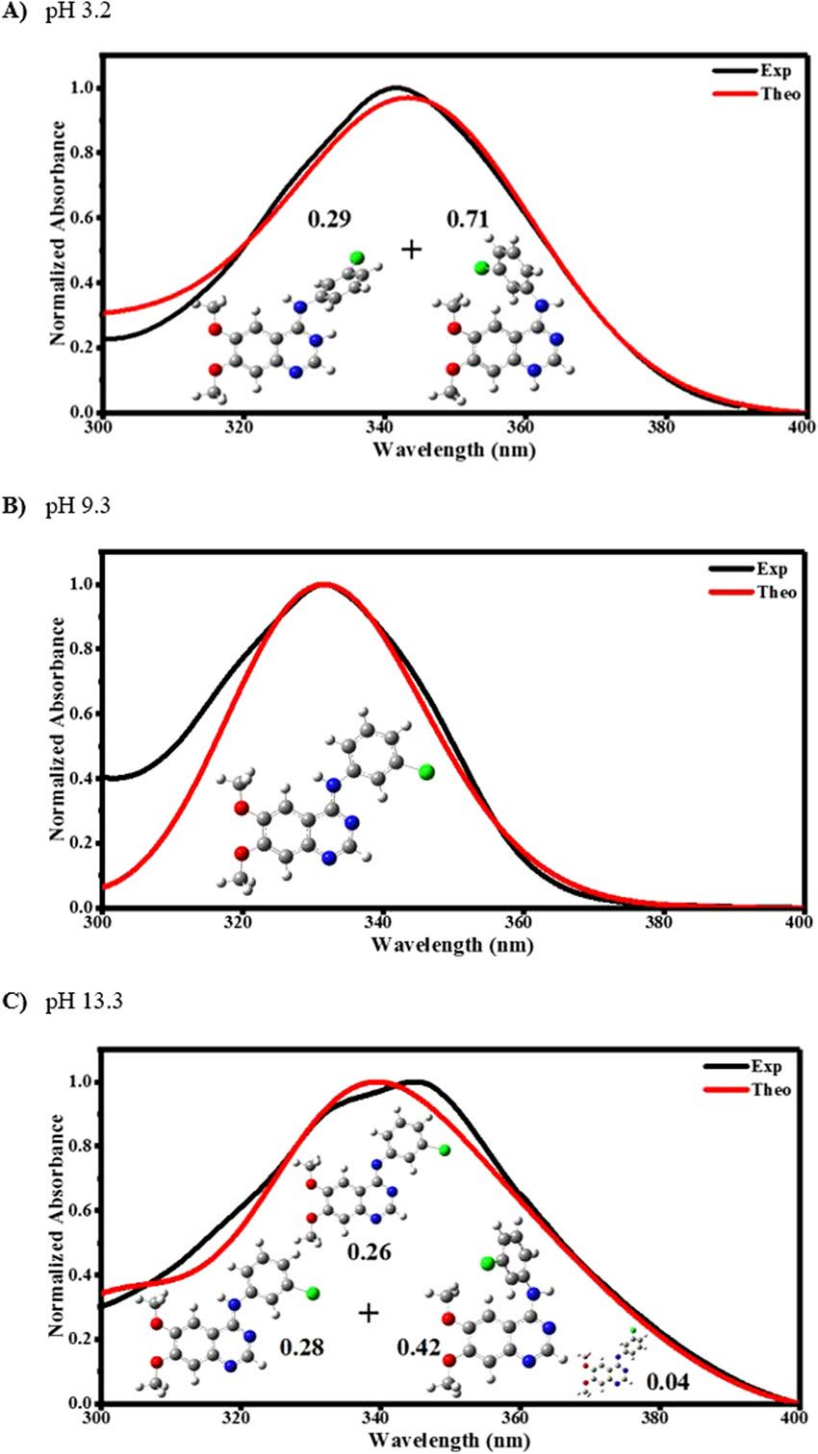
Fits of theoretical spectra to experimental spectra of AG1478 in (**a**) acidic (pH 3.2), (**b**) alkaline (pH 9.3) and (**c**) alkaline (pH 13.3) solution. White, grey, blue, red and green spheres denotes hydrogen, carbon, nitrogen, oxygen and chlorine atoms respectively.

To assign the experimental spectra, we exploited solver tool in excel to find the minimal value of sum of squared residuals between observed spectrum and theoretically fitted spectrum within the 300–400 nm region. In Fig. 6, the normalised experimental spectrum of AG1478 in acidic (pH 3.2), alkaline (pH 9.3) and strong alkaline (pH 13.3) solutions are depicted along with the best fit to the sum of theoretical spectra within region of 300–400 nm. To fit theoretical spectra to experiment at pH 3.2, the neutral and deprotonated forms of AG1478 were excluded since they cannot experimentally exist at low pH (AG1478 pKa = 5.6). The diprotic structures were excluded from the fit because the calculated diprotonated structures exhibited two transitions at *ca*. 360 nm and 324 nm while the experiment showed only one absorption maximum at 342 nm. Thus, monoprotic forms of AG1478 were only used to fit the experimental spectrum. We found that the combination of 71% of monoprotonated AG1478 (N1-LM2) and 29% of monoprotonated AG1478 (N3-LM1) accounted for the AG1478 spectrum at pH 3.2. The two structures adopted twisted conformations as can be seen in Fig. 6a.

The absorption maximum of AG1478 at pH 9.3 was observed at 332 nm while the lowest energy transitions of the deprotonated structures were calculated at 371 nm, 368 nm and 386 nm. We therefore excluded the anionic forms of AG1478, especially the plateaued AG1478 absorbance at pH 7.2–12 (Fig. 4a) indicated presence of only one prototropic state. The absorption maximum of GM and LM structures of neutral AG1478 were computed at 331 nm and 337 nm respectively. However, only the neutral planar (GM) conformer was reasonably fit to the experimental spectrum at pH 9.3 as shown in Fig. 6b.

The observed spectrum of AG1478 at pH 13.3 exhibited two absorption maxima at 334 and 345 nm. From the pH titration plot in Fig. 4b, we have a mixture of species at pH 13 (transition region). The calculated absorption maxima lie within 331–337 nm and the 368–386 nm for neutral and deprotonated structures respectively. Therefore, the experimental spectrum was fit to a mixture of neutral and deprotonated species. A mixture of neutral (GM), neutral (LM), deprotonated (GM) and deprotonated (LM2) forms contributed collectively by 28%, 42%, 26% and 4% respectively to the measured spectrum as indicated in Fig. 6c. Note that the deprotonated LM1 form was considered in the fitting to the spectrum but had a negligible contribution (<0.1%). It is noteworthy that the twisted neutral conformation (Neut-LM) was 1.5 fold more populated than the planar configuration (Neut-GM). However, the planar structure of deprotonated AG1478 contributed by more than 6-fold compared to its twisted counterpart.

## Discussion

By studying the pH-dependent spectral properties of the tyrosine kinase inhibitor AG1478 and combining these observations with theoretical calculations, we obtained new insights into acid/base interactions and geometrical/electronic configurations of the drug. Based on theoretical calculations, we identified the protonation sequence of AG1478 nitrogens and how it is correlated to experiments. The acid dissociation constant of the AG1478 molecule was determined from the experimental pH-titration curve. Combined experimental and theoretical studies enabled us to assign each experimental spectrum to the relevant contribution of AG1478 structures. We also identified structural conformations of AG1478, whether the aniline and quinazoline moieties are coplanar or twisted, at different pH values which has not been reported so far.

AG1478 contains three potential protonation sites at two quinazolyl nitrogens and amino moiety. At pH 0.3–3.2, the absorption maximum of AG1478 in 300–400 nm region was observed at 342 nm. The observed spectrum at pH 3.2 was assigned to two monoprotonated structures of AG1478 adopting twisted configurations. One structure is protonated at N(1) and the other one is protonated at N(3) and both contributed by 71% and 29% respectively. The absorption spectra of AG1478 exhibited a systematic dependence on solution acidity at pH 3.2–7.3. Starting from pH 7.3 to 12, the absorption peak was observed at 332 nm. The spectrum obtained at pH 9.3 was solely assigned to neutral planar conformer of AG1478. At pH 13 and 13.3, emergence of two overlapping bands were observed at 334 nm and 345 nm. The measured spectrum at pH 13.3 was assigned to the sum of neutral (planar (28%) and twisted (42%)) and deprotonated (planar (26%) and pseudo-planar (4%)) forms of AG1478. These results indicated that the coplanarity of aniline and quinazoline rings are favoured for neutral and deprotonated forms of AG1478 in alkaline solutions, while AG1478 adopts twisted configurations in acidic solutions.

The results of our study both complement and extend earlier studies on related molecules. Gefitinib, a 4-anilinoquinazoline-based tyrosine kinase inhibitor^57,58^, has the same chromophore and biological function as AG1478. The UV-Vis absorption measurements revealed that the diprotonated form (protonation at *only one* quinazolyl nitrogen) of Gefitinib prevails in acidic solutions of pH ≤ 3, giving rise to an absorption band at 340 nm. The neutral form of Gefitinib predominates in alkaline solutions at pH > 7.2 showing a blue-shifted absorption peak at 331 nm^59^. These results are in a good agreement (±2 nm) with our experiments. The absorption maxima of AG1478 in the acidic (pH 0.3–3.2) and alkaline (pH 7.2–12) solutions were 342 nm and 332 nm respectively. In addition, the acid dissociation constant of AG1478 (pKa = 5.6) was very close to that of Gefitinib (pKa = 5.4)^59^.

Our spectral assignments agree well with conclusions based on experimental and theoretical studies of quinazoline. In acidic conditions quinazoline consists of a mixture of monocations protonated at N1 or N3 and this has been rationalised theoretically by the similar protonation affinities at N1 and N3^56^. According to our experiments and theory N1 and N3 are both significantly populated at acid pH and have similar protonation affinities. Our assignment of neutral AG1478 at neutral to slightly alkaline pH agrees well with experimental studies on quinazoline^56^. The lack of di-protonated AG1478 or mono-protonated (^+^NH_2_ linker) AG1478 populations in our spectral assignments is consistent with the very low pKa for formation of these protonation states in the parent chromophores (pKa (di-protonated quinazoline) =−5.5^60–63^, pKa(^+^NH_2_ aniline) < 0)^64^.

Our results have potential implications for the pharmacodynamics of quinazoline-based drugs. Iressa®, Tarceva®, Tykerb®, Tyverb®, Gilotrif® and Caprelsa® are oral dosage forms of 4-aminoquinazoline-based tyrosine kinase inhibitors. Our results point to the prototropic and geometric forms of AG1478 during its journey in the gastrointestinal tract. According to our analysis, the *monocationic twisted* structure of AG1478 would be mainly populated in the gastric juice (pH = 2) of the stomach and duodenum (pH = 4.6). The *neutral planar* form of AG1478 would be the dominant (uncomplexed) drug species in the blood plasma and intestinal fluids (pH 7.4–7.6).

The cellular and sub-cellular environments of cancer cells can also vary in pH and this may influence AG1478 conformations, drug-cell interactions and cellular dynamics. In this context, the changes in pH of the microenvironment as the molecule transits from the vicinity of a cancerous cell (acidic) in the outer membrane to the nuclear interior (slightly basic) also gains relevance. For example, cancer cell membranes tend to be negatively charged, and so would be expected to preferentially bind the positively-charged AG1478 as opposed to the neutral AG1478. The twisted versus planar conformer of AG1478 might also confer different membrane binding, membrane translocation and intracellular trafficking properties. These speculations await further experimental enquiry. Overall, our study paves the way for understanding the conformation of anticancer drugs in different environments.

## Conclusion

By combining theory with experiment we have identified the conformations and protropic forms of AG1478 across the pH range 2.3–13.3 for the first time. The electronic absorbance spectrum of AG1478 was found to be an excellent reporter of the pH of its microenvironment and undergoes significant pH-induced transitions in amplitude and spectral position. Our calculations reveal that the structure of AG1478 undergoes a transformation from planar to twisted upon solution acidification. Overall, our results have ramifications for drug formulation and for understanding pharmacokinetics in the different pH environments encountered in the body and in cells.

## Materials and Methods

### Materials

*N*,*N*-Dimethylsulfoxide, phosphoric acid, boric acid, acetic acid and sodium hydroxide were purchased from Sigma Aldrich Pty Ltd. AG1478 was obtained from AdooQ Bioscience company. Millipore deionized water was used in preparation of universal buffer solutions ranging from pH 2.3–12. Universal buffer was prepared by adding equal volumes of 0.04 M phosphoric acid, 0.04 M boric acid and 0.04 M acetic acid and titrating the solution with 0.2 M NaOH to the required pH. The strong acidic (pH ≤ 2) and alkaline (pH ≥ 12) solutions were prepared by using 1 M HCl and 1 M NaOH, respectively. pH was measured using Mettler Toledo SevenEasy S20 pH meter to ± 0.01 pH resolution. A pair of matched quartz cuvettes of 1 cm path length was deployed in all experiments.

## Methods

### UV-Vis spectroscopy

Absorption measurements were conducted as described earlier^53^. Equal volume of buffer solution was added to both sample and reference cuvette. Since AG1478 is partially soluble in water, it was preferred to use another vehicle to deliver AG1478 into buffer solution and without affecting the characteristics of bulk aqueous environment. Therefore, we added 10 *μ*L of 2 mM AG1478 in DMSO to the sample cuvette and 10 *μ*L of pure DMSO to the reference cuvette. The final solution contained 10 *µ*M AG1478 in 99.5% (aqueous buffer): 0.5% DMSO (v/v). Solutions were shaken and left 10 minutes for equilibrium. Absorption maxima were obtained using originlab software. Values of mean, standard deviation and confidence interval were determined using Excel. Regression analysis of experimental spectrum was done using solver function in Excel. The observed absorption maxima were calculated at 95% confidence interval.

### Computational details

Relaxed potential energy surface scan was performed for four prototropic structures of AG1478 in vacuum using B3LYP/6–31 G model, the same model employed in our previous study^55^. The potential energy surface was built by varying N(12)-C(8)-N(25)-C(14) dihedral angle from 0° to 360° in 10° stepwise rotation. Density functional theory (DFT) and time-dependent DFT were deployed for geometry optimization of the ground state and for excitation energy calculations of excited state structures, respectively. Becke three-parameters Lee-Yang-Parr hybrid functional (B3LYP)^65,66^ in combination with 6–311 + G(d) basis set was employed in all other calculations. The calculated vibrational frequencies showed that all re-optimized structures are true local minima. The conductor-like polarizable continuum model (CPCM)^67^ with *ε* = 78.35 was used to approximately describe the polarity of bulk environment. The UV-Vis absorption spectra of different ionized AG1478 states in water were then calculated for the singlet–singlet transitions of the lowest 45 excited states. All simulations were performed using GAUSSIAN 09 Revision C.01^68^ on swinburne supercomputing facilities.

## Electronic supplementary material

Supplementary Information
