# Supplementary material for: A pH-induced conformational switch in a tyrosine kinase inhibitor identified by electronic spectroscopy and quantum chemical calculations
Source: Sci Rep. 2017 Nov 24;7:16271. doi: 10.1038/s41598-017-16583-z (PMC5701190; doi:10.1038/s41598-017-16583-z)

A pH-induced conformational switch in a tyrosine kinase inhibitor identified by electronic spectroscopy and quantum chemical calculations

Muhammad Khattab[a], Feng Wang[b,c,d*] and Andrew H. A. Clayton[a*]

[a] *Centre for Micro-Photonics, Faculty of Science, Engineering and Technology, Swinburne University of Technology, Melbourne, Victoria, 3122, Australia*

[b] *Molecular Model Discovery Laboratory, Department of Chemistry and Biotechnology, Engineering and Technology, Swinburne University of Technology, Melbourne, Victoria, 3122, Australia*

[c] *School of Chemistry (Bio21 Institute), University of Melbourne, Parkville, Victoria 3052, Australia*

[d] *School of Physics, University of Melbourne, Parkville, Victoria 3052, Australia*

*Corresponding authors: aclayton@swin.edu.au

fwang@swin.edu.au

Supplementary Information

Table S1. Molecular coordinates of optimised protonated structures of AG1478 using B3LYP/6-311+G* model

N(1)N(3)-GM

C	-0.00401400	-0.00456400	0.02425200
C	0.09236100	0.34471200	1.36471800
C	1.34382400	0.39703300	1.97091400
C	2.52735100	0.10507000	1.25489300
C	2.42057200	-0.24104000	-0.10884800
C	1.18842200	-0.30103000	-0.72778600
H	-0.79244900	0.57387100	1.94178800
C	3.77245300	0.16538900	1.95582700
H	3.30474500	-0.46147900	-0.68834200
C	2.60184400	0.80397600	3.93765000
H	2.65029100	1.07686300	4.98146300
N	3.73214100	0.52594200	3.28369000
N	1.45287900	0.74190600	3.31685200
C	6.21802200	-0.05618900	2.12396100
C	6.87929100	1.16229700	2.26382000
C	6.75847000	-1.24669400	2.60964100
C	8.10920200	1.18582300	2.91884600
H	6.44502800	2.06948500	1.86128700
C	7.98960900	-1.19138400	3.25374200
H	6.23062600	-2.18369400	2.48582700
C	8.67133800	0.01248100	3.41561700
H	8.63745800	2.12439700	3.03688400
H	9.62895800	0.03243700	3.92093100
Cl	8.69315000	-2.67472100	3.87461600
N	4.95377300	-0.10757100	1.42571100
H	4.97941900	-0.40221000	0.45813400
O	-1.13905100	-0.09371900	-0.65566600
O	0.98085200	-0.62012000	-2.00788000
C	2.10469900	-0.94313100	-2.84355900
H	1.68134900	-1.16751200	-3.81830500
H	2.78314300	-0.09150200	-2.92216200
H	2.63141100	-1.81842600	-2.45845100
C	-2.39133700	0.16957200	0.00692600
H	-3.15114200	0.02595100	-0.75531900

H	-2.54525500	-0.53624900	0.82452400
H	-2.41962000	1.19693700	0.37279600
H	4.60321100	0.57753000	3.80573400
H	0.61328600	0.95275900	3.84859000

N(1)N(3)-LM1

C	0.00000000	0.00000000	0.00000000
C	0.00000000	0.00000000	1.38866019
C	1.21124281	0.00000000	2.07339637
C	2.44919629	0.00072084	1.39112924
C	2.43991939	0.00204104	-0.01957566
C	1.24932181	-0.00303133	-0.71765038
H	-0.92861078	-0.00165814	1.94203293
C	3.64844415	0.00945681	2.16930853
H	3.36710830	0.01287501	-0.57295097
C	2.33125301	0.00097185	4.16279177
H	2.30496536	-0.00172813	5.24249757
N	3.51188594	0.00616905	3.53890540
N	1.22402641	0.00108025	3.46679486
C	6.09652001	0.06606742	2.43854272
C	6.83877519	-1.09982145	2.61443678
C	6.52301484	1.29304365	2.94707903
C	8.03509365	-1.03136597	3.32455961
H	6.48563839	-2.03783088	2.20360053
C	7.71745485	1.32726104	3.65993434
H	5.94508254	2.19354297	2.78180300
C	8.48020699	0.17895224	3.85287282
H	8.62576402	-1.92788650	3.47095507
H	9.40935137	0.22876759	4.40694982
Cl	8.27316428	2.85811476	4.31410576
N	4.87408212	0.01649488	1.67018082
H	4.97270045	-0.03578095	0.66449459
O	-1.08828460	0.00368196	-0.75764540
O	1.13375121	-0.00778572	-2.04805413
C	2.32190114	-0.02662090	-2.85677498
H	1.96884987	-0.04120089	-3.88380405
H	2.91983278	0.87029027	-2.68406861
H	2.90979735	-0.92399485	-2.65444712
C	-2.39024812	0.01580006	-0.13953396
H	-3.09579774	0.01841516	-0.96480527
H	-2.53165370	-0.87867502	0.46865563
H	-2.51738095	0.91678813	0.46228068
H	4.35021918	0.00061827	4.11379495
H	0.34296072	0.00346484	3.97191490

N(1)N(3)-LM2

C	0.85644300	0.13940000	-0.17661300
C	0.33598600	0.69492800	0.98467300
C	1.13308000	0.79257100	2.11882900
C	2.48199600	0.34770100	2.13234100
C	2.99010200	-0.23248100	0.94631300
C	2.21176900	-0.34123600	-0.18753400
H	-0.68537800	1.04760600	1.01987400
C	3.22184000	0.48503900	3.35126200
H	3.99875300	-0.60579000	0.92569100
C	1.26929500	1.41855800	4.39491400
H	0.81675900	1.82716100	5.28646800
N	2.53846300	1.01501000	4.43368500
N	0.58834700	1.32404000	3.28409900
C	5.53472800	-0.21728000	2.68237900
C	5.98643400	-1.53390000	2.67213000
C	6.13351700	0.77175400	1.90329500
C	7.05228300	-1.87092400	1.84029700
H	5.50882200	-2.27811400	3.29773600
C	7.18949300	0.40288500	1.07687300
H	5.78672600	1.79646500	1.94429800
C	7.65750700	-0.90796600	1.03546200
H	7.41387900	-2.89205200	1.81598700
H	8.48398200	-1.17159100	0.38724800
Cl	7.95162700	1.62634100	0.07387300
N	4.48478200	0.15823500	3.59925300
H	4.79153800	0.22133700	4.56462500
O	0.19176800	-0.00038900	-1.31418900
O	2.60650200	-0.87509400	-1.34815800
C	3.94590000	-1.37994900	-1.47083700

H	4.02019400	-1.74951800	-2.48959400
H	4.67300100	-0.58149500	-1.31279800
H	4.11541900	-2.19741200	-0.76766300
C	-1.17575000	0.44672800	-1.40510800
H	-1.47749900	0.22965600	-2.42526100
H	-1.80355000	-0.10620500	-0.70509900
H	-1.23903300	1.51952200	-1.21789300
H	3.02323100	1.13816300	5.31747700
H	-0.37664300	1.64149100	3.28498600

N(1)-GM

C	0.00252200	0.00019600	0.00061400
C	-0.00083500	0.00016900	1.38336400
C	1.21473200	-0.00002700	2.07898100
C	2.45304500	-0.00020700	1.40586900
C	2.43873500	-0.00014600	-0.00969200
C	1.25234800	0.00004600	-0.71041900
H	-0.92947600	0.00030600	1.93779100
C	3.64014600	-0.00043000	2.22805100
H	3.36265100	-0.00022400	-0.56958000
C	2.38149600	-0.00016200	4.13920100
H	2.30821000	-0.00011000	5.21980900
N	3.56356300	-0.00035800	3.57424900
N	1.23128500	-0.00002900	3.46089500
C	6.16909100	-0.00085500	2.18624300
C	7.21108500	-0.00104300	1.24529400
C	6.47045800	-0.00080800	3.55386800
C	8.53420600	-0.00118000	1.66453400
H	6.98567700	-0.00107900	0.18389300
C	7.80771500	-0.00094400	3.93710900
H	5.68599600	-0.00067000	4.29140600
C	8.85313200	-0.00113000	3.02240600
H	9.32845000	-0.00132400	0.92680600
H	9.88330600	-0.00123400	3.35535100
Cl	8.17828300	-0.00088000	5.66197000
N	4.85818200	-0.00072700	1.65038100
H	4.85527400	-0.00088300	0.64142200
O	-1.09137100	0.00037000	-0.76858900
O	1.14202400	0.00011800	-2.05118600
C	2.33631800	-0.00002000	-2.84315300
H	1.99918000	0.00006600	-3.87623700
H	2.93052700	0.89605800	-2.64989800
H	2.93027300	-0.89628100	-2.64996400
C	-2.38542600	0.00055500	-0.14567000
H	-3.09904700	0.00064000	-0.96485300
H	-2.52065500	-0.89581500	0.46268600
H	-2.52041100	0.89697900	0.46266000
H	0.35673800	0.00009900	3.97261800

N(1)-LM1

C	-0.06622000	-0.60845900	0.39563300
C	0.07770400	0.62555200	1.00337800
C	1.34750800	1.05126600	1.41340600
C	2.49182100	0.25154300	1.22251200
C	2.32715700	-1.01146100	0.60436000
C	1.08594400	-1.44511400	0.19266000
H	-0.77677000	1.26780200	1.16819100
C	3.74639600	0.79083600	1.68508900
H	3.17605000	-1.66239700	0.45350100
C	2.72058900	2.69479000	2.42528600
H	2.76507700	3.67107200	2.89262000
N	3.82093400	2.00023600	2.27802300
N	1.51495800	2.27884000	2.02528000
C	6.20842800	0.41874600	1.93563900
C	6.49449800	1.08487000	3.13099700
C	7.24915800	-0.02481600	1.11122900
C	7.82179100	1.31802200	3.48009300
H	5.69645400	1.41299200	3.77960600
C	8.56254400	0.21605100	1.49233500
H	7.03520400	-0.54514500	0.18523000
C	8.87153000	0.89028300	2.66864100
H	8.04355700	1.83429100	4.40736700
H	9.90200400	1.07087800	2.94794600
Cl	9.86567600	-0.34499700	0.44737600
N	4.88792900	0.09300200	1.52394000

H	4.82527000	-0.74429300	0.96453300
O	-1.22485000	-1.11857000	-0.03534000
O	0.83195900	-2.62529400	-0.40165600
C	1.92034600	-3.52510900	-0.64471500
H	1.47724400	-4.39269600	-1.12629200
H	2.65851300	-3.07164100	-1.31032200
H	2.39273500	-3.82649100	0.29307100
C	-2.43023000	-0.35593700	0.13140100
H	-3.22036600	-0.97048700	-0.29086600
H	-2.62795300	-0.17499300	1.18970500
H	-2.36690600	0.58940400	-0.41099900
H	0.71173900	2.87645900	2.17937200

N(1)-LM2

C	0.63714600	-0.42927400	0.33921600
C	0.25239100	0.10380800	1.55606900
C	1.21034800	0.69510100	2.38838300
C	2.57940700	0.72514700	2.03724100
C	2.94931200	0.20328900	0.77149200
C	2.01383300	-0.35591600	-0.06925800
H	-0.78327500	0.08953300	1.86682500
C	3.47120300	1.33312900	2.98824100
H	3.97700600	0.26251600	0.45366900
C	1.71436900	1.96162600	4.32395800
H	1.32454400	2.47423700	5.19489600
N	2.99619200	1.99438400	4.07491400
N	0.82714600	1.30729000	3.56484500
C	5.71443500	0.51807900	2.17762500
C	6.72322800	1.10073000	1.41025600
C	5.62873300	-0.87040600	2.30833300
C	7.64477600	0.28510600	0.75759600
H	6.77926000	2.17946800	1.32311300
C	6.55003400	-1.66001100	1.63103600
H	4.86550100	-1.32128300	2.92979300
C	7.56281800	-1.10258900	0.85552900
H	8.43001000	0.73250200	0.15913800
H	8.27342800	-1.73796400	0.34167800
Cl	6.44002700	-3.41072700	1.77891500
N	4.81603500	1.36166700	2.90025100
H	5.24032000	1.96417900	3.59693000
O	-0.18946800	-1.01208900	-0.53474700
O	2.26936400	-0.86106400	-1.29181200
C	3.61175000	-0.81229800	-1.78954700
H	3.57192800	-1.26522300	-2.77672700
H	3.95821000	0.22046500	-1.87238600
H	4.28547800	-1.38512500	-1.14848300
C	-1.58502900	-1.12335200	-0.21313700
H	-2.03585800	-1.63174400	-1.06080700
H	-1.72589100	-1.71662600	0.69245500
H	-2.03264400	-0.13486700	-0.09374400
H	-0.14864100	1.30733400	3.83648000

N(3)-GM

C	0.00938700	0.00900900	0.02842700
C	0.10438000	0.31296800	1.37343400
C	1.35391900	0.36233800	2.01443000
C	2.53004100	0.09430700	1.26853000
C	2.43030100	-0.21413500	-0.10912900
C	1.20138800	-0.25851300	-0.73061600
H	-0.77346700	0.51999100	1.96921900
C	3.77252900	0.15201700	1.97219800
H	3.31593800	-0.42099100	-0.69303500
C	2.52710300	0.71512200	3.93626000
H	2.62028100	0.95462500	4.98832900
N	3.71392100	0.46710600	3.28872800
N	1.37820800	0.67261100	3.36045900
C	6.23006600	-0.02310500	2.09615700
C	7.05393500	1.08767400	1.91964500
C	6.62198500	-1.09458800	2.90011500
C	8.29041900	1.12373900	2.55998100
H	6.72871600	1.90680400	1.28942600
C	7.85689100	-1.02610000	3.53728900
H	5.98262300	-1.96090600	3.01483300
C	8.69898500	0.07008700	3.37574800
H	8.94104300	1.98035000	2.42816200

H	9.65832200	0.10061900	3.87735900
Cl	8.36483200	-2.36544700	4.55636300
N	4.96490700	-0.08291200	1.41332800
H	4.98994100	-0.26098900	0.41984800
O	-1.13471100	-0.06394700	-0.66425500
O	0.99756400	-0.54066400	-2.03095300
C	2.12959400	-0.81853200	-2.86398200
H	1.72215600	-1.00761100	-3.85361000
H	2.80458800	0.03964200	-2.90233800
H	2.66434200	-1.70363900	-2.51152100
C	-2.37587900	0.17777200	0.01572000
H	-3.14684900	0.05919300	-0.74070700
H	-2.52593400	-0.55055300	0.81517500
H	-2.40477800	1.19221300	0.41816600
H	4.57463900	0.53266000	3.82107600

N(3)-LM1

C	-0.07398200	-0.49433300	0.42345100
C	0.13891400	0.61849200	1.21529300
C	1.43495500	0.97254100	1.62720000
C	2.53558600	0.17256500	1.22628600
C	2.31556200	-0.96082600	0.40816500
C	1.04201800	-1.30065000	0.00736900
H	-0.67901900	1.24725800	1.53814700
C	3.82915000	0.57080600	1.68364000
H	3.14451300	-1.57061500	0.07865800
C	2.76999000	2.41433200	2.78155100
H	2.95674900	3.28700800	3.39515400
N	3.88839400	1.68829000	2.44752500
N	1.57927500	2.10128400	2.41030800
C	6.27736500	0.29955400	1.82755500
C	6.90201400	1.39908200	1.23896000
C	6.92041400	-0.45767700	2.80776900
C	8.18603700	1.75089200	1.65092700
H	6.39807600	1.96148000	0.46159400
C	8.20689800	-0.09263400	3.18779400
H	6.42355500	-1.30792100	3.25780600
C	8.84881500	1.00724700	2.62467700
H	8.68071400	2.60332000	1.20026300
H	9.84988800	1.27593300	2.93857600
Cl	9.03393900	-1.04108200	4.41544800
N	4.95963700	-0.09106700	1.40817500
H	4.87832900	-0.97296800	0.92310400
O	-1.26993300	-0.90842300	-0.01476300
O	0.72674100	-2.35394900	-0.76938000
C	1.77825300	-3.20997200	-1.23258400
H	1.28799300	-3.97590700	-1.82763000
H	2.48464200	-2.65662600	-1.85579100
H	2.30007400	-3.67464300	-0.39276000
C	-2.44180600	-0.16648800	0.35590900
H	-3.27303900	-0.68926300	-0.10925700
H	-2.56768900	-0.16099900	1.44037300
H	-2.38798700	0.85565800	-0.02390400
H	4.78628900	1.99852100	2.80265100

N(3)-LM2

C	0.63530100	-0.39620800	0.33994700
C	0.27330100	0.24918100	1.50692100
C	1.23892900	0.85782600	2.32547200
C	2.61648800	0.77850000	1.96739100
C	2.97447700	0.14522300	0.75017100
C	2.01802800	-0.42984800	-0.05506600
H	-0.76019800	0.32229900	1.81519800
C	3.54806400	1.40678100	2.84689500
H	4.00582600	0.12653600	0.43953500
C	1.67293000	2.14668300	4.15076100
H	1.39018700	2.72730900	5.02019100
N	3.01863500	2.08988700	3.89797100
N	0.78984800	1.54841400	3.43059900
C	5.75565100	0.48412400	2.10583900
C	6.76183300	0.94595600	1.25826400
C	5.63547900	-0.87454200	2.40442800
C	7.65003400	0.03349100	0.69384300
H	6.84230600	2.00455800	1.04067300
C	6.52246000	-1.76404200	1.80958400

H	4.87330400	-1.22748500	3.08751600
C	7.53394400	-1.32998900	0.95731600
H	8.43461800	0.38499300	0.03384700
H	8.21780200	-2.04112300	0.51091700
Cl	6.36974100	-3.48043200	2.16557000
N	4.89090100	1.43689100	2.73457700
H	5.37213400	2.13130000	3.29305400
O	-0.21351700	-1.00007900	-0.49924400
O	2.25829400	-1.04338200	-1.22921600
C	3.60682300	-1.11335700	-1.70848200
H	3.55079600	-1.64400900	-2.65532700
H	4.01378900	-0.11280700	-1.87124300
H	4.23913300	-1.66867800	-1.01215200
C	-1.61484400	-1.01295000	-0.18409100
H	-2.08696500	-1.56224800	-0.99392900
H	-1.79203900	-1.52418000	0.76402500
H	-2.01050500	0.00368800	-0.14563500
H	3.63833800	2.53993500	4.56213600

Neut-GM

C	-4.00873800	-0.23157100	0.00029000
C	-3.35370600	-1.44275400	0.00005000
C	-1.93990300	-1.50699200	-0.00016800
C	-1.18796600	-0.30634800	-0.00011900
C	-1.87345300	0.93648000	0.00009900
C	-3.24942700	0.98787000	0.00029800
H	-3.89599800	-2.37864300	0.00001300
C	0.24344500	-0.46875300	-0.00027900
H	-1.32775200	1.87006000	0.00009100
C	-0.03097400	-2.74116400	-0.00061300
H	0.46204200	-3.71029700	-0.00084600
N	0.79720600	-1.67599500	-0.00054000
N	-1.34262000	-2.74132900	-0.00042400
C	2.44118000	0.79538500	-0.00026400
C	2.92864900	2.11557200	-0.00061500
C	3.35556100	-0.26858200	0.00003400
C	4.29332200	2.36529700	-0.00066100
H	2.23143200	2.94768700	-0.00084800
C	4.71621700	0.02145500	-0.00003300
H	3.00314200	-1.28658900	0.00028500
C	5.21546400	1.31678000	-0.00037500
H	4.64830400	3.39001700	-0.00093600
H	6.28149400	1.50568000	-0.00042500
Cl	5.85338200	-1.33307300	0.00035600
N	1.04422700	0.64481000	-0.00014900
H	0.55366800	1.52464200	0.00001400
O	-5.35015400	-0.07383800	0.00051500
O	-3.98751900	2.12377200	0.00050900
C	-3.30786300	3.38100400	0.00055500
H	-4.08968400	4.13667200	0.00075800
H	-2.69033900	3.49334200	0.89552900
H	-2.69060700	3.49355100	-0.89457800
C	-6.17385600	-1.24424000	0.00058200
H	-7.19845100	-0.88060700	0.00082100
H	-5.99656400	-1.84581600	-0.89406600
H	-5.99620000	-1.84596800	0.89505500

Neut-LM

C	2.89681300	-0.90709200	-0.01690100
C	3.14737300	0.35549700	0.47015100
C	2.18046400	1.38396600	0.35441200
C	0.91875900	1.09067500	-0.23025700
C	0.68685200	-0.20545300	-0.76290700
C	1.64392100	-1.18768400	-0.66798100
H	4.09226700	0.60644000	0.93258800
C	-0.00422700	2.18149900	-0.28078000
H	-0.24675400	-0.41535200	-1.26177200
C	1.60246500	3.57210800	0.57470500
H	1.86580000	4.58838000	0.85666600
N	0.35965500	3.41046700	0.08199400
N	2.51203200	2.64293600	0.77117000
C	-2.24809600	1.06724000	-0.54041000
C	-3.32065400	0.93986000	-1.43262800
C	-2.18278700	0.23580000	0.58436100
C	-4.30875300	-0.01178700	-1.20498900

H	-3.37113400	1.58467700	-2.30313600
C	-3.17200600	-0.72236300	0.77157000
H	-1.38297100	0.33971700	1.30570100
C	-4.24297600	-0.86547400	-0.10331900
H	-5.13590100	-0.10215400	-1.90044100
H	-5.00273000	-1.61753200	0.06842000
Cl	-3.07054000	-1.77343400	2.18654900
N	-1.28901900	2.07857600	-0.78430100
H	-1.68418300	2.98830000	-0.98873100
O	3.75612400	-1.94568400	0.04117600
O	1.52230000	-2.44668600	-1.15295400
C	0.31403000	-2.80178900	-1.82910200
H	0.43721300	-3.84140100	-2.12307600
H	-0.54920500	-2.70800300	-1.16542600
H	0.16742100	-2.18430800	-2.71908200
C	5.02963100	-1.74589300	0.66329900
H	5.53917900	-2.70416700	0.59989300
H	5.60785600	-0.98568700	0.13272500
H	4.91140300	-1.46007300	1.71117700

De-GM

C	0.02460200	-0.01407100	0.00715300
C	0.00997200	0.01329500	1.38701300
C	1.21573600	0.03175100	2.13100100
C	2.44807000	0.02062200	1.44537400
C	2.45089200	-0.00818600	0.03223800
C	1.27430000	-0.02486000	-0.68656800
H	-0.92116400	0.02213500	1.93889800
C	3.67980800	0.04025200	2.23913200
H	3.40987300	-0.01649800	-0.46581000
C	2.31480700	0.07800600	4.12007100
H	2.27707100	0.10240400	5.21018600
N	3.55017600	0.06917200	3.59848400
N	1.14590500	0.06077900	3.50790100
C	6.09347900	0.03138900	2.13281900
C	7.17360600	0.09980700	1.21101000
C	6.44382800	-0.04810500	3.50943900
C	8.49759500	0.09248600	1.62119300
H	6.93218800	0.15868500	0.15459100
C	7.78143000	-0.05599100	3.88197000
H	5.66278700	-0.09846800	4.25039900
C	8.83391100	0.01377700	2.97846300
H	9.29012100	0.14761800	0.88090800
H	9.86499300	0.00735600	3.30938800
Cl	8.16559900	-0.16046800	5.62063100
N	4.83155500	0.03627700	1.57591800
O	-1.08341000	-0.03210600	-0.78283600
O	1.18766400	-0.05122100	-2.04970300
C	2.40245600	-0.05741600	-2.79545700
H	2.10770900	-0.07662300	-3.84272100
H	2.99252900	0.84237500	-2.59857700
H	3.00222600	-0.94361300	-2.56865300
C	-2.36334900	-0.02068400	-0.15165000
H	-3.09142700	-0.03727100	-0.95979600
H	-2.49934000	-0.90194900	0.48107700
H	-2.50078000	0.88480300	0.44567500

De-LM1

C	0.08054700	-0.60886900	0.03848700
C	0.03488200	0.23566300	1.12955700
C	1.22275800	0.68658900	1.75714000
C	2.46768000	0.26030000	1.25106200
C	2.50411700	-0.60203000	0.13233300
C	1.34444900	-1.03717200	-0.47404900
H	-0.90867900	0.57578900	1.53641700
C	3.67951800	0.72703000	1.92584300
H	3.47422200	-0.90931700	-0.23251900
C	2.28299200	1.89006900	3.36906300
H	2.22151300	2.56173000	4.22693900
N	3.52579300	1.55549300	3.00260200
N	1.12418500	1.52709200	2.84543600
C	6.05228700	0.58087300	2.08944800
C	6.26910300	0.39102500	3.47654800
C	7.18378200	0.90186100	1.30158100
C	7.53737500	0.51691300	4.03031700

H	5.42909300	0.13685400	4.10979900
C	8.43435100	1.03095800	1.88673300
H	7.05716500	1.04104000	0.23419200
C	8.64984200	0.84538900	3.24801100
H	7.67268200	0.35638200	5.09598100
H	9.63759900	0.94574500	3.68045900
Cl	9.81869800	1.44520200	0.84981000
N	4.85468300	0.36354700	1.43494600
O	-1.01109900	-1.08884900	-0.61798500
O	1.28851700	-1.86912700	-1.55681600
C	2.51971100	-2.32891500	-2.10837800
H	2.24943000	-2.96707400	-2.94725400
H	3.12993100	-1.49447500	-2.46617200
H	3.08949600	-2.90937400	-1.37689100
C	-2.30352100	-0.70289300	-0.15175800
H	-3.01474500	-1.19566700	-0.81117000
H	-2.46726600	-1.03558700	0.87686500
H	-2.43826900	0.38046000	-0.21291700

De-LM2

C	0.67556900	-0.33356600	0.34881700
C	0.28272700	0.14118600	1.58163600
C	1.20628000	0.76632700	2.45760700
C	2.56920800	0.85110200	2.08304800
C	2.94711600	0.39360500	0.79556200
C	2.03647000	-0.18176800	-0.06356400
H	-0.74713000	0.07524800	1.90750300
C	3.49552600	1.46879500	3.03726700
H	3.97278600	0.50775700	0.48073800
C	1.63240200	1.96185600	4.33873700
H	1.25991000	2.45604800	5.23671100
N	2.94380100	2.09123100	4.12421100
N	0.72931300	1.30691100	3.62719600
C	5.63694500	0.66026700	2.28465000
C	6.78625300	1.11970700	1.59416000
C	5.48163700	-0.74728600	2.40394600
C	7.70052800	0.23463500	1.03715500
H	6.93962900	2.19045900	1.50523100
C	6.41442100	-1.60027900	1.83786900
H	4.63504700	-1.14977400	2.94693400
C	7.53442900	-1.15022000	1.14413200
H	8.56532000	0.62313000	0.50756800
H	8.24623400	-1.84322200	0.71370200
Cl	6.17335100	-3.35467000	2.01665700
N	4.81429600	1.56053700	2.91073100
O	-0.14516800	-0.93636600	-0.55109000
O	2.32044000	-0.63466500	-1.32024500
C	3.65579900	-0.49090300	-1.79869700
H	3.65735200	-0.90912000	-2.80314500
H	3.94927500	0.56202900	-1.84180500
H	4.36271900	-1.04233500	-1.17238000
C	-1.51875400	-1.11127600	-0.20234800
H	-1.97705200	-1.61309700	-1.05168200
H	-1.62213400	-1.73375500	0.69049400
H	-2.00911700	-0.14807400	-0.03814700

Figure S1: Theoretical absorption spectra of studied AG1478 structures in water using B3LYP/6-311+G* model. All spectra are plotted using the same scale.

$^{+}$N(1)H^{+}N(3)H

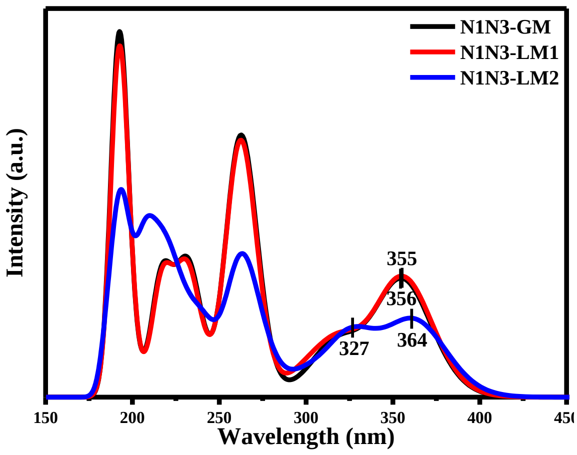

$^{+}$N(1)H

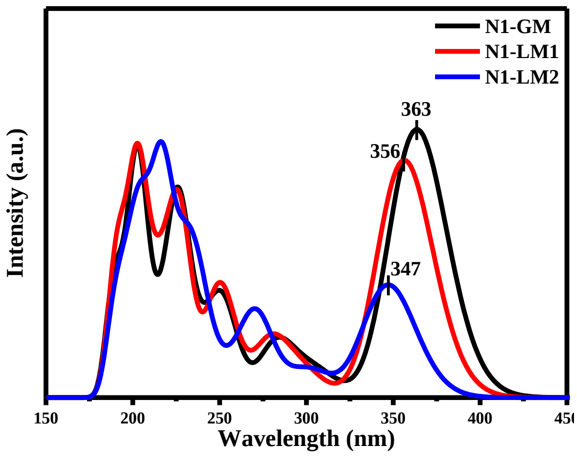

$^{+}$N(3)H

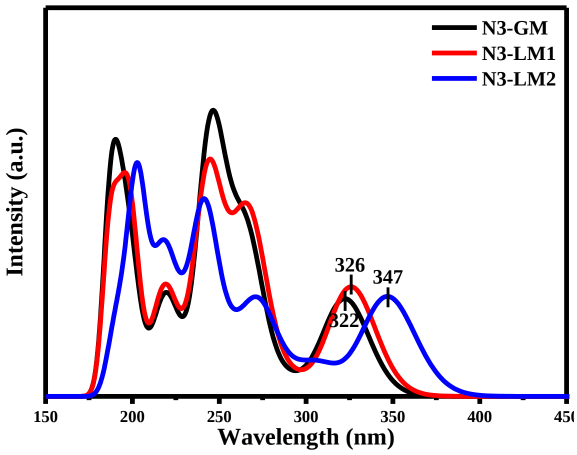

Neut.

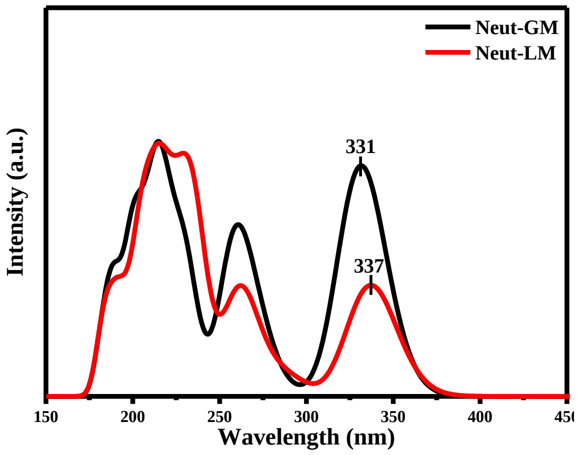

Deprotonated

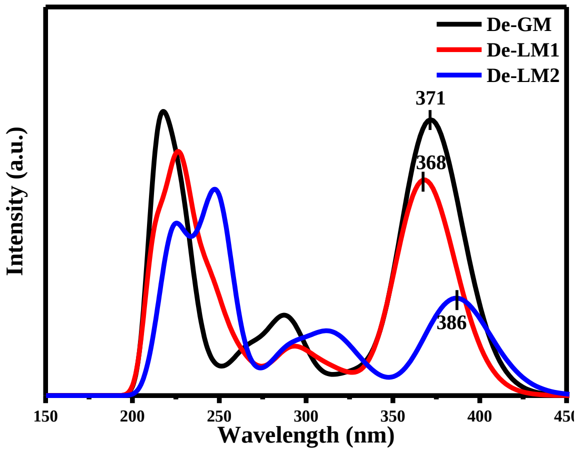

Supplement: Supplementary file 1 — Supplementary Information [file 41598_2017_16583_MOESM1_ESM.pdf]
